# Opportunities and Limitations of Modelling Alzheimer’s Disease with Induced Pluripotent Stem Cells

**DOI:** 10.3390/jcm3041357

**Published:** 2014-12-05

**Authors:** Dmitry A. Ovchinnikov, Ernst J. Wolvetang

**Affiliations:** Stem Cell Engineering Group, Australian Institute for Bioengineering and Nanotechnology, The University of Queensland, St. Lucia 4072, Australia; E-Mail: d.ovchinnikov@uq.edu.au

**Keywords:** induced pluripotent stem cells, Alzheimer’s disease, disease modelling, reprogramming

## Abstract

Reprogramming of somatic cells into induced pluripotent stem cells (iPSCs) has opened the way for patient-specific disease modelling. Following their differentiation into neuronal cell types, iPSC have enabled the investigation of human neurodegenerative diseases, such as Alzheimer’s disease (AD). While human iPSCs certainly provide great opportunities to repeatedly interrogate specific human brain cell types of individuals with familial and sporadic forms of the disease, the complex aetiology and timescale over which AD develops in humans poses particular challenges to iPSC-based AD models. Here, we discuss the current state-of-play in the context of these and other iPSC model-related challenges and elaborate on likely future developments in this field of research.

## 1. Opportunities and Limitations of Modelling Alzheimer’s Disease with Induced Pluripotent Stem Cells

The ability to generate patient-specific induced pluripotent stem cells (iPSCs) through reprogramming of somatic cells and, following their differentiation into neuronal cell types, investigate the aetiology of human neurodegenerative diseases, such as Alzheimer’s disease (AD), has created much excitement about this new *in vitro* disease modelling paradigm. While human iPSCs certainly provide great opportunities to repeatedly interrogate specific human brain cell types of individuals with familial and sporadic forms of the disease, the complex aetiology and timescale over which AD develops in humans poses particular challenges to iPSC-based AD models. Here, we discuss the current state-of-play in the context of these and other iPSC model-related challenges and elaborate on likely future developments in this field of research.

## 2. iPSCs as a Model System

Following the ground-breaking work by Takahashi, Yamanaka and others [1], the concept of personalized disease modelling with induced pluripotent stem cells, generated from a patient’s own somatic tissues, is now firmly established (e.g., see [2,3,4,5,6]). While larger cohorts of iPSCs from various diseases are being generated worldwide through various consortia, industry or iPSC banks, a survey of the literature indicates that the vast majority of studies are limited to the comparison of a few disease and control samples (Table 1). While this in no way invalidates the data obtained thus far, there is evidence that iPSCs, even from the same individual, can vary in terms of both DNA mutation load [7], gene expression [8] and epigenetic signatures [9,10,11,12,13,14,15], often resulting in differences that may affect their propensity to differentiate into particular cell types [16]. Others, however, report no or only a few differences in gene expression between different hESC and iPSC lines [17,18,19]. Some of the variability appears to be driven by the method chosen to reprogram the somatic cells, with non-integrating methods showing the least variability [20,21,22], allelic variation [23,24], the age and type of cells used for reprogramming [25] and the culture time and method used to expand iPSC following establishment [26]. Rarely, researchers have shown, however, that three independent clonal iPSC lines from multiple patients with the same disease statistically differ from controls and that this does not change with increased passage number. Given what we now know about the erosion of imprinting at affected loci, as well as the variability (and erosion) of X-chromosome inactivation [27,28], parameters that can profoundly affect neurally-differentiated cell types, these are important factors to consider when embarking on or interpreting iPSC disease modelling studies. Similarly, the issue of choosing the appropriate controls for comparative studies of human samples is not a trivial one. While unaffected sibling or parental control samples are preferable, these are not always available or come from family members of different age or gender and different genetic make-up. We predict that with time, there is likely to be an increasing demand for the isogenic gene-corrected controls (if the mutation is known [4]) or verification of the causality of single or compounded disease-associated alleles through the introduction of such mutations into control (“disease-unaffected”) iPSC lines through genome editing technologies (e.g., using CRISPRs (Clustered Regularly Interspaced Short Palindromic Repeats) or TALENs (Transcription Activator-Like Nucleases) [29,30]), thereby reducing the need for very large (and costly) disease and patient-specific iPSC cohorts.

**Table 1:** iPSc models of Alzheimer’s disease.

Genetic Defect	Affected Process(es)	Disease Type, iPS/hES *N* and *n* *	Transgene-Free?	Investigated Cell Type(s)	Reference(s)
*APP*	Aβ production and aggregation, MAPT	Familial early-onset (*N* = 2, father + daughter; *n* = 2 pre-selected)	N	Neurons	[31]
*APP*	Aβ production, ER stress	Familial early-onset (*N* = 2, *n* = 2 and 3) and sporadic (*N* = 2; *n* = 2)	Y	Cortical neurons, astrocytes	[32]
*PSEN1*	β-amyloid processing	Early-onset AD, OE model in *N* = 1 hES and *N* = 1 iPS	Y/N	Neurons	[33]
*PSEN1*, *PSEN2*	β-amyloid processing	Early-onset AD, *N* = 2 *PSEN1&2*; *n* = 2	N	Neurons	[34]
*ApoE(4)*	Aβ levels	Early and late-onset DA, familial (*N* = 2) and sporadic (*N* = 3)	N	Basal forebrain cholinergic neuron	[35]
*PSEN1*	Aβ production and aggregation, MAPT	Familial AD, *N* = 4	N	Neural stem cells, neurons	[36]
*APP* and *PSEN1 OE*	Aβ production and processing	OE models of familial AD mutations	N	Neural precursor cells, neurons	[37]

OE, Overexpression; * *N*, Number of analysed individuals (unrelated, unless stated otherwise), *i.e.*, population size; *n*, Number of independently-generated iPS clones, *i.e.*, sample size, N = No; Y = Yes; Y/N = Undetermined.

## 3. Making the Right Cell Type

**Figure 1:**
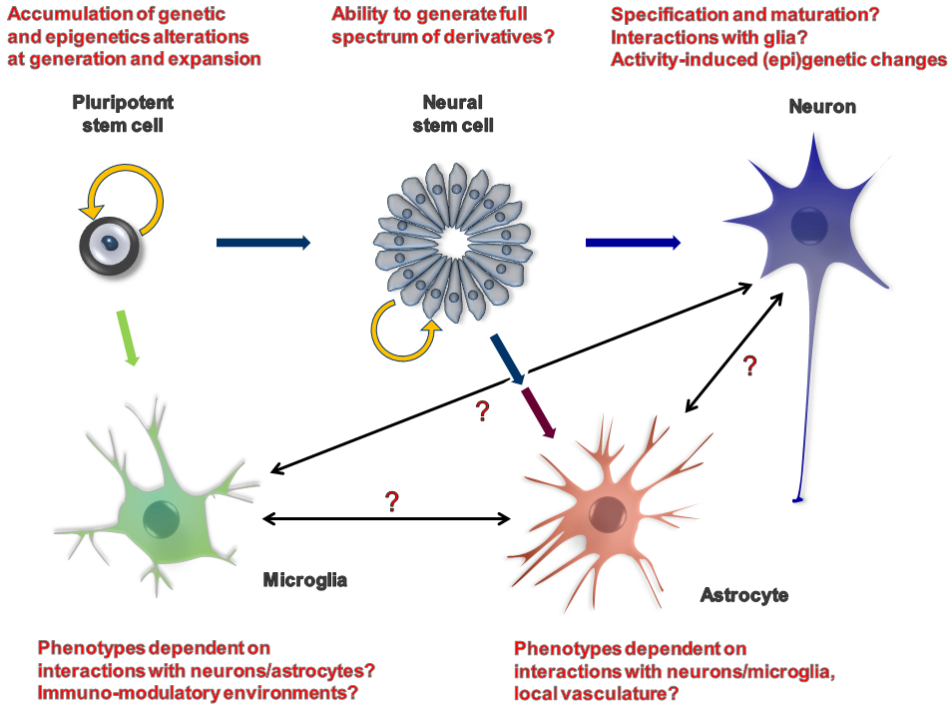
Modelling Alzheimer disease with iPSC-derived cell types has the potential to reveal cell-cell and paracrine signalling events underlying disease aetiology.

AD is characterized by progressive dementia accompanied by the occurrence of neuritic plaques (NP), mainly comprised of extracellular deposits of amyloid beta (Aβ) protein and neurofibrillary tangles (NFT), consisting of intracellularly-aggregated hyperphosphorylated tau protein [38]. With the exception of familial forms of the disease, constituting approximately 2%–5% of disease burden, the vast majority of clinically seen AD is the sporadic form of the disease, and despite many decades of research, its aetiology remains largely enigmatic. Sporadic AD can vary in its time of onset, severity and clinical read-outs and may in fact encompass multiple AD-like diseases with distinct aetiologies. Glutamatergic and basal forebrain cholinergic neurons in the cerebral cortex and the hippocampus are thought to be cells that are affected at early stages and lost during AD pathogenesis, with further loss of GABAergic and other neuronal cell types during the advanced stages of the disease [39]. These AD tell-tale signs further appear to be invariably associated with, and perhaps driven by, astrocyte and microglial activation, as well as changes in local vasculature [40]. Given that AD development is clearly a gradual process involving the interaction of multiple cell types in a complex three-dimensional milieu and typically first observed in specific regions of the ageing brain, what is the correct iPSC-derived cell type that will most faithfully model AD *in vitro* (Figure 1)? Thus far, most iPSC-AD modelling studies have employed either embryoid body/neurosphere or small molecule-based neuronal differentiation protocols that are known to generate mainly glutamatergic cortical forebrain neurons [32,33,34,41,42,43]. In terms of gene and neuronal marker expression, these largely cortical neuronal cultures at 4–9 weeks still consist of a mixture of different cell types of variable maturity levels, most closely resembling early human foetal neurons (a conclusion largely based on gene expression and functional analyses of their action potentials and calcium-handling ability). Despite these facts, and perhaps as a testament to the robustness and expressivity of certain AD phenotypes, increased Aβ42 amyloid production and tau-phosphorylation changes have been observed in such cultures. There is a clear need, however, to develop protocols that will allow the generation of specific and relevant cell types (e.g., basal forebrain cholinergic neurons) and purify such neurons away from differently patterned neuronal cell types if we are to decipher the gene-regulatory networks involved in disease initiation. There is a similar need for the development of protocols that will mimic or accelerate the maturation and “ageing” processes of such neurons *in vitro*. Researchers have started to explore this concept through subjecting neural cells to prolonged culture [44], the transient delivery of progerin [45], telomere shortening [46], chronic exposure to oxidative stress [47], DNA damaging agents [48] or proteasome inhibitors [49,50]. Similarly, the field has started to embrace the concept that AD is not a solely neuron-driven disease, but involves an interaction between neurons and astrocytes [51] (and likely microglia [52] and the local microvasculature [40]) that, while initially beneficial, upon reaching a certain threshold, becomes deleterious to neuronal function and survival [53]. Even though it is difficult to envisage that we will be able to artificially recreate such a complex, three-dimensional tissue as the human brain at this stage, iPSC technology is well suited to study paracrine interactions in the dish [54,55], particularly since astrocytes can be readily isolated from control or AD neuronal cultures using flow cytometry or magnetic bead technology and co-cultured with neurons from control or AD patients. Experiments of this type recently identified astrocytes as an important contributor to neuro-degeneration in Down syndrome iPSC-derived neuronal cultures, a condition that displays AD with a 100% penetrance [56]. Adding microglia, the third cell type of the AD pathogenesis “triad”, to such an *in vitro* model is now achievable. While differentiation of microglia from mouse pluripotent stem cells is achievable [57,58,59], the generation of this yolk sack haematopoiesis-derived macrophage cell type [60] from human pluripotent stem cells has thus far not been reported. The biggest advantage of any iPSC-based AD modelling exercise will remain the ability to gene-edit the cells by the introduction of the specific mutations or transgenes and corroborate the causality of newly-discovered cell-cell or gene-gene interactions. Combining such an approach with single-cell sequencing technology may be the key to uncovering whether increasing cellular heterogeneity, occurring over time, and possibly induced by normal neuronal activity, is a contributing factor in AD pathogenesis.

## 4. AD Phenotypes Which Can be Reliably Modelled *in Vitro*

**Figure 2:**
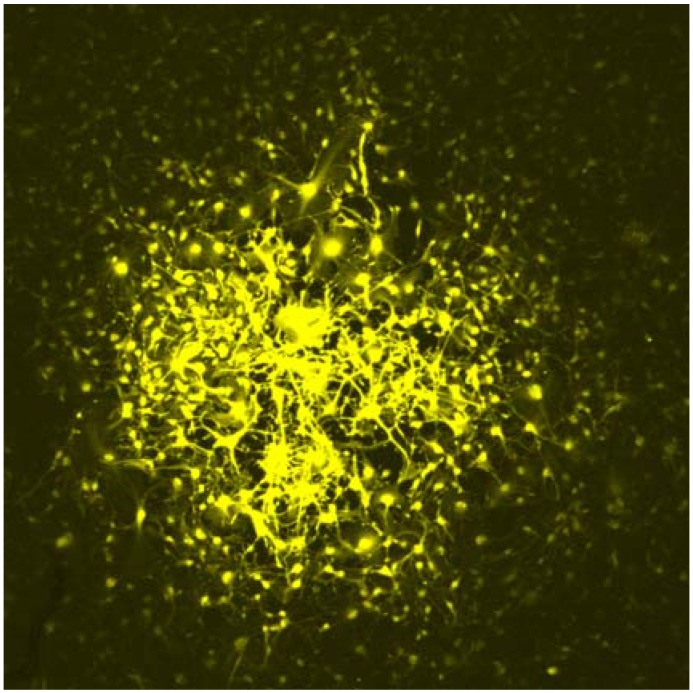
A Day 70 neuronal culture from control iPSCs, imaged 30 min after one cell was micro-injected with NeuroBiotin™ and detected using Streptavidin-Cy3, reveals the highly interconnected nature of neurons and astrocytes generated *in vitro*. Image courtesy of Patrick Fortuna and Refik Kanjhan (University of Queensland, Brisbane, Australia).

There appears to be forming an increasing consensus that AD-like pathological changes involve early alterations in phosphorylation of the neuronal protein, tau, and its aggregation into neurofibrillary tangles (NFTs), followed and exacerbated by beta-amyloid toxicity and plaque formation [61,62,63]. In AD patients, cognitive decline correlates closely with the decreased thickness of cortical layers in various regions of the brain and predicts progression to AD [64,65]. At a superficial level, subjecting AD iPSC-derived neurons to cell survival assays before and after noxious stimuli, such as oxidative stress, appears sensible and is, thus, commonly used [66]. A correlate that closely matches the cognitive decline in AD is the occurrence of NFTs, rather than beta-amyloid plaques [67]. Determining the level of the microtubule-associated protein, tau, the main constituent of NFT, and the prevalence and subcellular localisation of its different phosphorylated forms would therefore seem essential, since tau appears to act as a key mediator or enabler of both Aβ- and apoE4-dependent AD pathogenesis. In one study [34], the expression of tau or phospho-tau isoforms was not observed, whereas others [41] did observe this in familial and one sporadic AD-iPSC-derived neurons [31]. Measuring the activity and phosphorylation status of GSK3β, one of the key kinases involved in tau-phosphorylation [68], is also commonly a part of the analysis [69]. Although it has become clear that the role of β-amyloid in AD pathogenesis is much more complex than was initially appreciated, with perhaps early neuro-protective roles for APP and clear neuro-degenerative effects of aggregated processed forms, such as Aβ42 during later stages, measurements of the expression of APP and its processed forms remains a highly relevant parameter to examine. Indeed, elevated levels of extracellular Aβ42, as well as the presence of intracellular aggregates have been reported in iPSC-based models of AD [32]. There is further increasing evidence that APP and β-amyloid are linked to enlargement and altered localisation of early endosomal compartments marked by RAB5, and this has indeed been reported in AD iPSC-derived neurons [41]. Both tau and β-amyloid have been linked to a loss of dendritic spines and synapses in mice and humans, and this is another parameter that closely matches the cognitive decline in AD [70]. While these can be readily measured in neurons generated *in vitro* from AD-iPSCs, this approach has so far been under-used, perhaps owing to the fact that identification and binning of different neuronal subtypes is still difficult to achieve. Notably, there is evidence in mouse models of AD that synapto-dendritic degeneration is often preceded by an aberrant neuronal network activity [71,72], suggesting that inappropriate synaptic wiring or network stimulation may be an early contributor to AD pathogenesis. While rabies virus-based synaptic connectivity assays have been used to good effect in iPSC models of schizophrenia [73] and neuronal connectivity in the dish can be readily assessed through dye injection (Figure 2, [74]), these have thus far not been used to any extent in iPSC-based AD research. Given emerging evidence that neuronal activity may stimulate retrotransposon mobility [75], induce double-stranded DNA breaks [76], elicit epigenetic changes in neurons [77,78,79,80,81,82] and trigger the expression of activity-dependent long non-coding RNAs (lncRNAs) [83,84,85], this may provide a fertile “hunting ground” for finding novel AD-linked pathogenic mechanisms.

## 5. Down Syndrome iPSC as a Model for AD

All individuals with Down syndrome develop an early-onset AD. An obvious candidate gene for this phenomenon is APP (amyloid precursor protein), which resides on chromosome 21. Although increased *APP* gene dosage can certainly be a major driver of AD, as indicated by the fact that families with *APP* gene duplications develop early onset AD [86,87,88] and the lack of discernible AD pathology in partial trisomy 21 patients lacking the gene [89], this does not mean that other *HSA21* genes do not contribute to AD in DS. Indeed, mouse models and clinical data clearly indicate important AD enhancing roles for the DYRK1A kinase (because of its ability to directly phosphorylate tau, APP and RCAN1) [90,91,92], RCAN1 (a calcium regulated phosphatase able to increase tau-phosphorylation through inhibition of the phosphatase calcineurin and to regulate vesicle fusion kinetics) [93,94], ETS2 (a transcription factor upregulated by oxidative stress that transactivates APP) [95,96,97] and BACE2 (a non-amyloidogenic θ-secretase) [98,99,100].

Since the genetic defect in Down syndrome is known (trisomy 21), iPSCs from DS individuals present an attractive model to test hypotheses of AD pathogenesis. Indeed, we and others have shown that neuronally-differentiated DS iPSCs exhibit a number of phenotypes akin to AD, including: Increased neuronal cell death that can be rescued by anti-oxidants, reduced neurite extension numbers [66], reduced synapse formation, increased Aβ42 production and hyperphosphorylated tau [42]. DS iPSCs subjected to neural differentiation also show enhanced gliogenesis, generating astrocytes that exhibit an activated phenotype and increased ROS production levels, upregulation of iNOS, yet reduced expression of NFE2L2, TSP-1 and TSP-2, consistent with the reduced neuroprotective and neurotrophic ability of such astrocytes [56]. It is therefore evident that DS iPSC-derived neural cell types recapitulate key features of AD. Importantly, we and others were able to isolate isogenic euploid iPSC from reprogrammed DS fibroblast cultures, providing the ideal isogenic controls needed for gene regulatory network analysis. Advanced genome interrogation tools, such as CRISPR, can now be used to delete specific genes or gene cohorts on chromosome 21, and delivery of XIST to HSA21 has already been used to epigenetically silence the supernumerary trisomy 21 genes [101]. We anticipate that such genome modifying technologies in iPSC will rapidly provide novel insights into the cell-autonomous and non-cell autonomous processes underlying AD pathogenesis in DS. This will be of great relevance to understanding the bases of the sporadic AD in the general population. The time is now ripe for testing the effect of susceptibility loci identified through GWAS studies, such as ApoE ε4 allele PICALM, BIN1, SORL1, clusterin/ApoJ and CR1 [102] using, for example, CRISPR technology in iPSC models of AD disease and testing their contribution to *in vitro*-assessable phenotypes.

## 6. Drug Screening Utilizing AD iPSC-Derived Cell Types

AD iPSC-derived neurons are currently being used to screen for drugs that could be of potential benefit to patients. Encouragingly, compounds that inhibit gamma-secretase activity were effective at reducing beta-amyloid production in AD iPSC-derived neuronal cultures. Non-steroidal anti-inflammatory drugs, such as sulindac sulphide, show effectiveness in presenilin 1-overexpressing cells, albeit not for the L166P mutant [43]. Similarly, minocycline was able to normalize the pathological phenotypes of DS astroglia [56], emerging as a promising drug candidate for DS-associated AD and possibly familial AD, as well. In order to enable the high-throughput capability required for screening large chemical libraries, the field will need to address the issue of identifying, generating and culturing the correct cell types (discussed above), make informed choices about what cellular readout will be most informative in terms of preventing early AD changes in the brain and consider the fact that a combination of drugs will affect multiple cell types that are functionally inter-linked to the disease process, providing challenges to image analysis and culture platforms alike. A recent study by Choi *et al.* [37] demonstrated that the generation of three-dimensional cultures of familial AD-recapitulating human neurons was essential and sufficient to reproduce some aspects of the AD phenotype, such as extracellular amyloid-β plaque and neurofibrillary tangle formation. While the study did not utilize the iPS cells per se, the multipotent neural progenitor cell line used closely resembles neural progenitor cells generated during standard neural iPS differentiation [66].

## 7. Conclusions

Although it is still a relatively young field of research, the iPSC-based disease modelling of AD has made great progress in a short time, and it is anticipated that, as more AD researchers come to appreciate both the value and limitations of this platform, exciting new discoveries that will ultimately benefit dementia patients are likely to be forthcoming. Recent advances in footprint-free iPSC generation, single cell and epigenome analysis technology and the ability to introduce or correct combinations of sequence variants in iPSC are set to accelerate this process.
